# Calibrated automated thrombogram II: removing barriers for thrombin generation measurements

**DOI:** 10.1186/s12959-021-00312-8

**Published:** 2021-08-28

**Authors:** P. L. A. Giesen, A. J. W. Gulpen, R. van Oerle, H. ten Cate, M. Nagy, H. M. H. Spronk

**Affiliations:** 1 CoagScope BV, Maastricht, The Netherlands; 2 Departments of Internal Medicine and Biochemistry, Cardiovascular Research Institute Maastricht (CARIM), Maastricht University, Maastricht, The Netherlands

**Keywords:** Thrombin generation, Calibrated automated thrombogram (CAT), ST®-Genesia, Direct oral anticoagulants (DOACS)

## Abstract

**Background:**

Thrombin generation (TG) assessed by Calibrated Automated Thrombogram (CAT-I) reflects the overall capacity of plasma to generate thrombin, thus evaluating the balance between the anti- and procoagulant processes. However, with this method the calibrator curve is usually not measured until completion which has a severe impact on the calculation of the TG parameters, especially under conditions where almost all substrate is consumed. In addition, direct thrombin inhibitor (DTI) cannot be present in the calibration sample due to inhibition of the calibrator. We have developed a modified TG assay (CAT-II) and performed head-to-head comparison with the CAT-I method using the same fluorometer. Furthermore, we have compared our CAT-II method to a new automated TG instrument (ST®-Genesia) using the same calibration method.

**Methods:**

TG was assessed with CAT-I and CAT-II using the same fulorometer and with ST®-Genesia in control plasma and plasma containing different anticoagulants (dabigatran, rivaroxaban, apixaban) and plasmas to which common interfering substances, bilirubin, hemoglobin and lipids were added. In CAT-I, calibration was against the same plasma containing calibrator in the presence of fluorogenic substrate (Z-GGR-AMC). In contrast, CAT-II method and ST®-Genesia used a standard concentration of thrombin in buffer and 7-amino-4-methylcoumarin (AMC) in a separate plasma sample for calibration.

**Results:**

TG obtained from CAT-I using anticoagulant-free plasmas was lower compared with TG from CAT-II but both methods demonstrated an intra-assay variation less than 5% on all measured parameters. When comparing the two different calibration methods in the presence of different anticoagulants, a high correlation was seen in the presence of rivaroxaban and apixaban (R^2^ > 0.97), but not with dabigatran, a direct thrombin inhibitor. CAT-II method showed dose-dependent inhibition of TG in the presence of dabigatran, while CAT-I was not able to detect it. Both methods were able to correct for the interfering substances.

**Conclusions:**

Our results showed high similarity between the results of CAT-I and CAT-II method when it is applied in control plasmas and plasmas not inhibited with a direct thrombin inhibitor. Furthermore, both the CAT-II method and ST-Genesia using the same calibration method were able to detect the effect of all oral anticoagulants. Taken together, applying a new calibration method is a significant improvement for monitoring patients on direct thrombin inhibitors while not introducing any bias to results obtained on other types of samples.

## Introduction

The Calibrated Automated Thrombogram (CAT-I) method was developed and designed for the measurement of thrombin generation in plasma [1]. CAT-I has been proposed as a potential diagnostic test to elucidate the risk of hemorrhage or thrombosis in patients with or without anticoagulant treatment [2–4].

In CAT-I, thrombin generation is assessed in citrated plasma, that is recalcified and triggered by tissue factor and phospholipids, through recording of the increase in fluorescent signal due to cleavage of a fluorogenic substrate (Z-Gly-Gly-Arg-AMC) by thrombin [1]. In order to estimate the amount of thrombin formed, this change in fluorescent signal is compared to thrombin activity in another sample of the same plasma containing a known concentration of thrombin in complex with alpha-2-macroglobulin (α2M-T); the so-called thrombin calibrator [1]. There are several artefacts that can interfere with the calibration process and as a consequence with the final thrombin generation results. First, the same amount of AMC added to different plasma samples does not result in the same amount of fluorescent signal due to the variability of color between the different samples. This is why each plasma sample requires its own calibration. Second, the relationship between the AMC concentration and fluorescent signal is not linear, it bends off due to the so-called inner filter effect. Third, the velocity by which thrombin converts substrate depends on the substrate concentration which lowers significantly during the experiment. This means that the velocity of increase in fluorescence is not linear with the concentration of thrombin [1].

**Fig. 1:**
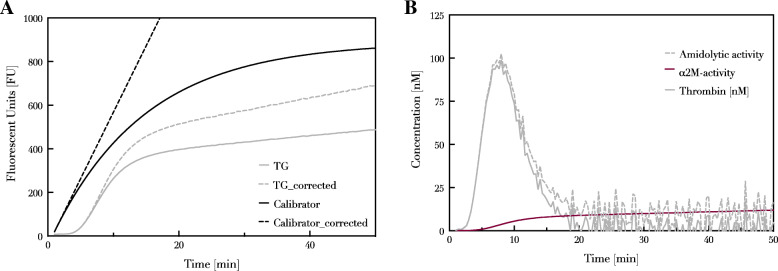
The process of obtaining thrombin generation curve. **A** Continuous lines show the raw fluorescent data obtained from the measurement of the calibrator and from thrombin generation whilst the dotted lines represent the curves corrected for inner filter effect and substrate consumption. **B** First derivative of the corrected signal represents the thrombin activity

The existing CAT-I method aims to overcome these problems by applying mathematical corrections [5] using calibrator measurements. The measured fluorogenic signal (FU) in the TG sample is then obtained and converted into thrombin concentration (nM). This conversion from FU/min to the concentration of thrombin is derived from the initial slope of the calibration curve that provides the link between the known concentration of thrombin (depending on the fluorimeter, 100 nM thrombin usually corresponds to about 20–50 FU/min) and FU/min. The calibrator curve bends off due to the inner filter effect and the substrate consumption, therefore the fluorescence curve obtained from measuring the calibrator is turned into a straight line and the parameters needed for this correction are then applied to the thrombin generation curve by using a mathematical correction (Fig. 1A).

After this correction, the first derivative of the signal is taken providing the total amount of amidolytic activity expressed as thrombin (nM) formed (Fig. 1B). However, the obtained TG curve still contains the residual amidolytic activity of α2M-thrombin complex (α2M-T) that is formed during thrombin generation (Fig. 1B). This α2M-T complex has no physiological function anymore but it is still able to convert the fluorogenic substrate [5]. In order to obtain the final TG curve, a mathematical correction is applied to remove the residual amidolytic activity.

Apart from the artefacts mentioned above, CAT-I has also some drawbacks that may affect the outcome. First, the calibration requires a long time to complete and usually the calibration is not measured until completion resulting in the omission of the plateau phase. This has the consequence that this part of the curve needs to be extrapolated by the software program in order to make the appropriate mathematical corrections to adjust the TG measurement. Second, the conversion of substrate by α2M-T may be different from the conversion by free thrombin which may have an impact on the proper corrections. Last but not least, the calibrator is measured in the same plasma as in which TG is measured and the presence of direct thrombin inhibitors (e.g. dabigatran) will inhibit the α2M-T complex [6] rendering the assay unsuitable for monitoring thrombin generation in plasma of patients on direct thrombin inhibitors.

A solution has been found in a new CAT-II method which overcomes the problems mentioned above by modifying the calibration of the measurements. First of all, the thrombin required for the calibration is measured in buffer containing a known concentration of free human thrombin. For the TG measurement in plasma, the citrated plasma sample is divided into two separate subsamples. To the first sample, trigger (tissue factor and phospholipids) is added, and thrombin generation is initiated by the addition of the fluorogenic substrate (Z-Gly-Gly-Arg-AMC) and calcium chloride (FluCa), identical to the procedure of the CAT-I method. However, to the second subsample a known amount of the fluorophore AMC is added. The latter will provide a fixed amount of fluorescence that is used to correct for the donor-dependent variation in color of the plasmas. The ratio between the fluorescence from AMC in plasma samples and in the buffer is used to provide a virtual calibration curve that is no longer dependent on the color of the medium. The calibration curve generated this way is used to correct for the artefacts mentioned above. Because the calibrator is no longer measured in plasma (as in the previous CAT-I method) direct thrombin inhibitors do not interfere with the calibration and the calibrator curve, which is now a measurement of thrombin in buffer, is always measured until completion.

Understanding the difference between CAT-I and CAT-II methodology becomes important as a recently developed fully automated thrombin generation analyzer for clinical routine analysis, ST®-Genesia applies this new CAT-II methodology. ST®-Genesia has been shown to have high reproducibility due to the precise temperature control and the normalization of TG parameters against a well-defined reference plasma [7, 8].

In this study, we propose this alternative calibration approach allowing the measurement of thrombin generation in patients receiving direct thrombin inhibitors. Furthermore, a careful comparison is done between CAT and ST®-Genesia. The aim of the current study was to describe this modified method and investigate its applicability for monitoring patients on direct thrombin inhibitors.

## Methods

### Blood collection and plasma preparation

Blood was drawn from healthy volunteers after a full informed consent in accordance with the declaration of Helsinki. Blood was collected into 3.2%(w/v) trisodium citrate. Platelet poor plasma (PPP) was obtained by centrifuging the citrated whole blood twice at room temperature (2500 x g for 5 min and 10,000 x g for 10 min). Plasma samples from 12 healthy volunteers were pooled together and aliquots were stored at − 80 °C until use.

### Thrombin generation using the CAT-I method

Plasma thrombin generation was performed according to our in house standardized operating procedure [9]. In brief, PPP samples were thawed for 10 min at 37 °C and used for measurement or supplemented with various procoagulants (Feiba®, Cofact®, Aafact®), direct oral anticoagulants (DOACs; rivaroxaban, apixaban or dabigatran), interfering substances (hemoglobin, bilirubin, lipid), or vehicle when is indicated. Samples were divided into two subsamples, the first subsample (80 μL) was supplemented with 20 μL of calibrator and the second subsample (80 μL) was added to 20 μL of trigger-reagent containing tissue factor (TF) and phospholipids (PL) (STG-ThromboScreen). Thrombin generation was assessed in clotting plasma upon addition of fluorogenic substrate for thrombin (Z-Gly-Gly-Arg-AMC) plus calcium chloride (FluCa). Thrombin generation was recorderd by measuring the signal of the cleaved AMC at 37 °C using a Fluoroskan Ascent (Thermofisher) plate reader with the wavelengths set to 390 (excitation)- 460 nm (emission) using a 96-well plate. Samples were measured in quadruplicates. The thrombin measured over time was calculated with the Thrombinoscope (Diagnostica Stago, Asnières-sur-Seine, France) software version 5 and the following parameters were derived: lag time (initiation phase of coagulation), endogenous thrombin potential (ETP; area under curve), peak height (PH; maximum level of thrombin generated), time to peak and velocity index (rate of thrombin generation until the PH).

### Thrombin generation using the CAT-II method

PPP samples were handled and treated as described in the CAT-I method above. Calibration was performed in the buffer system by measuring the fluorescence derived from a known concentration of thrombin and fluorogenic substrate (AMC) in buffer. This calibration was performed only once. Samples were divided into two subsamples, the first subsample (80 μL) was supplemented with 20 μL of AMC and the second subsample (80 μL) was added to 20 μL of trigger-reagent containing TF and PL (STG-Thromboscreen). The measurement was initiated by addition of 20 μL fluorogenic substrate (Z-Gly-Gly-Arg-AMC) plus calcium chloride (FluCa). Thrombin generation was assessed at 37 °C by measuring the signal of the cleaved AMC using a Fluoroskan Acsent (Thermofisher) plate reader with the wavelengths set to 390 (excitation)- 460 nm (emission) in a 96-well plate. All samples were measured in quadruplicates. The thrombin measured over time was calculated with a modified Thrombinoscope (Diagnostica Stago, Asnières-sur-Seine, France) taking into account the modified calibration. Thrombin generation parameters obtained are the same as listed with CAT-I method.

### Thrombin generation using the ST®-Genesia system

The ST®-Genesia (Diagnostica Stago, Asnières-sur-Seine, France) is a fully automated thrombin generation analyzer using individual cuvettes for measurements thereby allowing the performance of singular testing. Measurements were done as described for the CAT-II method. A known concentration of thrombin solution (STG-ThrombiCal) is divided into two subsamples, to one subsample fluorogenic substrate (STG-FluoStart) is added and fluorescence is measured in time until all substrate is converted and to the other subsample a fixed amount of AMC (STG-Fluoset) is added, and fluorescence is measured for a few minutes. This measurement of thrombin solution is performed only once prior to the start of the measurements in plasma.

For thrombin generation measurements in plasma, the samples were also divided into two subsamples: to one subsample STG-FluoSet is added and fluorescence is measured for a few minutes and to the other subsample a trigger (STG-ThromboScreen) and STG-FluoStart is added and fluorescence is measured until completion of the thrombin generation curve. All measurements take place in duplicate at 37 °C and the thrombin mediated substrate cleavage is measured at 377 (excitation)- 450 nm (emission) wavelengths. The thrombin measured over time is calculated by the software built-in in the ST®-Genesia using the same calculation algorithm as the modified Thrombinoscope software applied with CAT-II method. Thrombin generation parameters obtained are the same as listed with CAT-I method.

### Statistics

All statistical analyses were carried out using the IBM® SPSS® Statistics version 23. Coefficient of Variability (CV) was used for expression of the reproducibility. One-way ANOVA test was used for evaluating the statistical differences between CAT-I and CAT-II. Significant differences were considered when *p* < 0.05. Bland-Altman plot was used to assess the difference between different methods. For graphical and statistical purposes, GraphPad Prism (Version 8.4; GraphPad Software Inc.) was used. Data are presented as mean [±SD].

## Results

### Comparable intra-assay variation with CAT-I and CAT-II assays

In order to compare the repeatability and precision of CAT-I and CAT-II assays, intra-assay coefficients of variation (CV%) were calculated using 7 thrombin generation curves derived from a non-treated plasma sample. All parameters derived from thrombin generation, e.g. lag time, ETP, peak height and time to peak showed a between-run variation of less than 5% (Table 1). However, significant differences occurred between the two methods. Thrombin generation measured by CAT-I method was significantly delayed indicated by longer lag time and time to peak (*p* = 0.0005 vs *p* < 0.0001, respectively) and the overall thrombin generation was lower indicated by lower ETP and peak height (*p* < 0.001 vs *p* = 0.018, respectively) in comparison with the CAT-II method.

**Table 1:** Mean CAT parameters and %CV measured using CAT-I and CAT-II method

TG parameter	CAT-I		CAT-II		***P***-value
	Mean [SD]	CV%	Mean [SD]	CV%	
Lag time (min)	2.65 [0.06]	2.4	2.35 [0.04]	1.9	0.0005
ETP (nM.min)	1284 [49.1]	3.8	1402 [68.32]	4.9	0.0147
Peak height (nM)	246.6 [6.64]	2.7	265.9 [9.59]	3.6	0.018
Time to peak (min)	5.26 [0.13]	2.5	4.98 [0.04]	0.8	0.0195

*TG* thrombin generation; *CAT* calibrated automated thrombogram; *CV* coefficient of variation, *ETP* endogenous thrombin potential

### Difference in the effect of drugs using two different methods

**Fig. 2:**
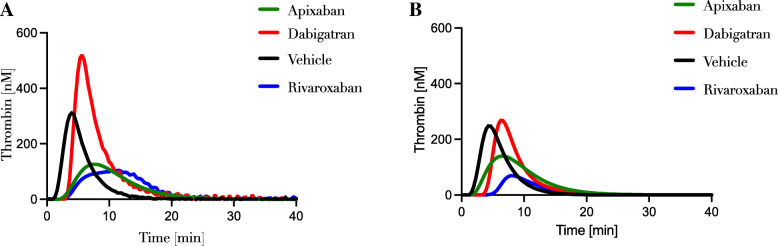
Thrombin generation curves in the presence of various DOACs. Thrombin generation was obtained from plasma spiked with vehicle or 62.5 ng/ml apixaban, dabigatran or rivaroxaban. **A** Thrombin generation curves assessed by using the CAT-I method. **B** Thrombin generation curves obtained by using CAT-II method

Given the difference between the calibration methods in CAT-I and CAT-II, the correlation coefficient between parameters was calculated to assess similarity. Thrombin generation was measured in pooled plasma of 12 healthy individuals and spiked with increasing concentrations of pro- or anticoagulant substances. The presence of procoagulant substances (0–3 U/mL Aafact® (Factor VIII concentrate), 0–2.5 U/mL Cofact® (prothrombinase complex), 0–3 U/mL Feiba® (prothrombinase complex with FVIIa)) showed more than 95% correlation between CAT-I and CAT-II (r ranged between 0.867–0.999), indicating that these factors do not interfere with the calibrator in CAT-I (data not shown). Similarly, the Factor Xa inhibitors apixaban and rivaroxaban (0–500 ng/mL final concentration) demonstrated high correlation (> 97% for all parameters) between CAT-I and CAT-II methods. On the other hand, measurements in the presence of the direct thrombin inhibitor dabigatran resulted in poor correlation between the two methods with the exception of lag time that appeared to have high similarity (99%). In contrast, peak height showed only a weak correlation (31%). These described differences are apparent from thrombin generation curves obtained from the two different methods (Fig. 2).

**Fig. 3:**
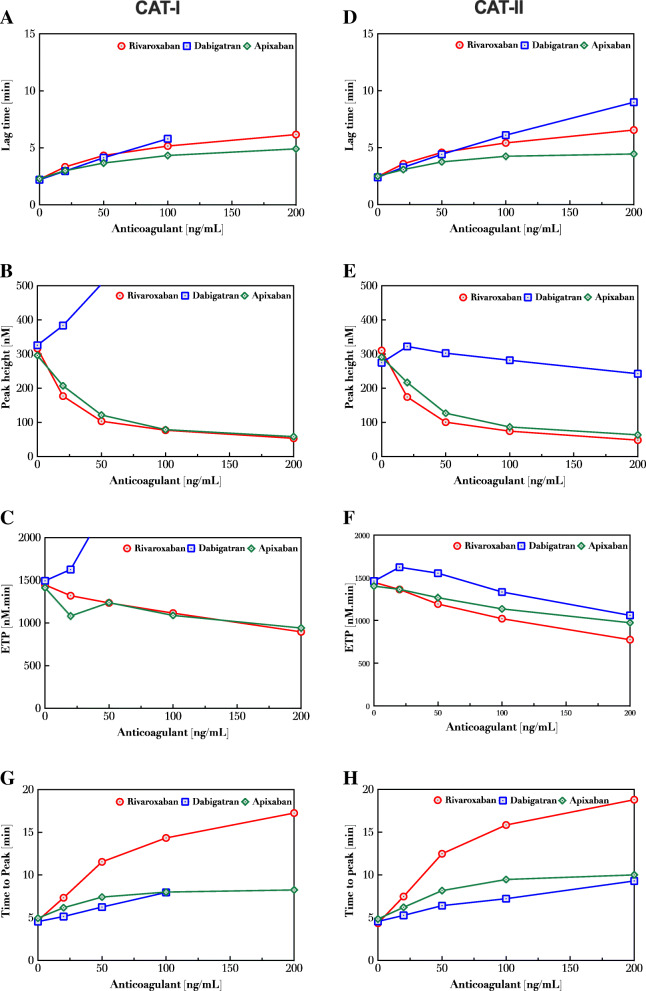
Dose-dependent effect of DOACs on thrombin generation. Plasma samples were spiked with dabigatran, apixaban or rivaroxaban (0–200 ng/ml) and thrombin generation was measured with CAT-I and CAT-II. Lag time (**A**,**D**), peak height (**B**,**E**), ETP (**C**, **F**) and time to peak (**G**,**H**) were determined in thrombin generation curves obtained with CAT-I and CAT-II methods

Given the variability seen between the different DOACs, we further investigated their effects on thrombin generation. Thrombin generation was measured in plasma samples spiked with different concentrations (0–500 ng/mL) of dabigatran, rivaroxaban and apixaban using both calibration methods. In thrombin generation assessed by CAT-I, rivaroxaban and apixaban dose-dependently decreased the ETP and peak height accompanied by a prolongation of the lag time (Fig. 3A-C) and time to peak (Fig. 3G-H). In contrast, the presence of dabigatran increased the peak height and ETP in a dose-dependent manner when thrombin generation was assessed with the CAT-I method using the α2M-T complex calibrator (Fig. 3A-C), while using the CAT-II method with the alternative calibration method, both ETP and peak height demonstrated a lower thrombin generation potential in a dose-dependent manner in the presence of either direct FXa or thrombin inhibitor (Fig. 3D-F). The results of the CAT-II method reveal that the modification of the calibration method indeed enables the detection of the inhibitory effect of dabigatran on the thrombin generation.

### Interference of various endogenous substances on thrombin generation

The variability between samples caused by endogenous or exogenous substances is a common problem and can often interfere with the absorbance of the plasma samples. These alterations resulting in reduced transmittance can alter the thrombin generation in CAT method by interfering with the calibration. Hence, we compared the sensitivity of the CAT-I and CAT-II method for the most common circumstances. Three endogenous compounds, hemoglobin (0–1.5 mg/mL), bilirubin (0–300 μM) and lipids (0–3 mM) were used for supplementation of the plasma samples in order to resemble the most common conditions like hemolysis, hyperbilirubinemia or lipidemia. Thrombin generation was then determined in these plasma samples using CAT-I and CAT-II method.

**Fig. 4:**
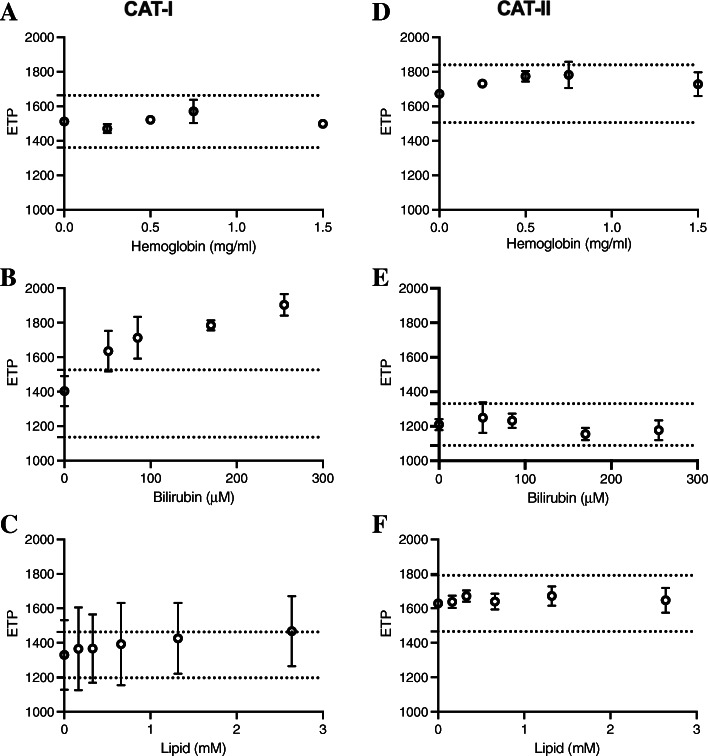
Interference of hemoglobin, bilirubin and lipids on ETP in CAT-I and CAT-II. Thrombin generation was measured in plasma samples supplemented with hemoglobin (0–1.5 mg/ml), bilirubin (0–300 μM) or lipid (0–3 mM). ETP was calculated from thrombin generation curves obtained in the presence of hemoglobin (**A**,**D**), bilirubin (**B**,**E**) or lipid (**C**,**F**). Data are expressed as mean ± SD. The dotted lines represent the ±2SD of the control ETP value

The analysis revealed different influences of the three substances on thrombin generation between CAT-I and CAT-II. The CAT-I assay was not influenced by hemoglobin (Fig. 4A), but bilirubin seemed to have a greater impact on the outcome, resulting in a dose-dependent mild elevation of ETP (Fig. 4B). Lipid supplementation increased the variability of the measurements (Fig. 4C). This effect was seen both for ETP (Fig. 4A-C) and peak height (data not shown). The ETP in the presence of both bilirubin (> 51 μM) and lipid (> 2.64 mM) was outside normal range, suggesting that these data are most likely due to an artefact caused by interference.

In contrast, the effects of all three substances were negligible in the CAT-II method as ETP measurements appeared within the normal range (Fig. 4D-F). Similarly, the peak height obtained from the CAT-II measurements were also within the 95% confidence interval (data not shown). These data suggest that the modified calibration method used in CAT-II method is able to correct for endogenous interferences present in plasma.

**Fig. 5:**
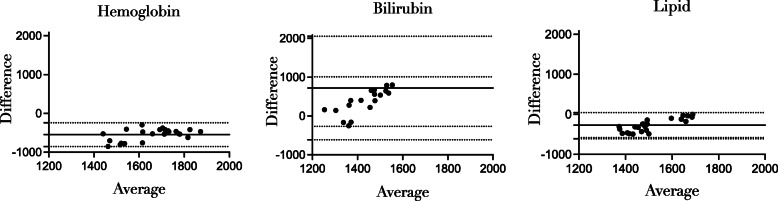
Difference between CAT-I and CAT-II method in presence of hemoglobin, bilirubin or lipid. ETP values obtained from CAT-I and CAT-II were compared in a Bland-Altman plot. The average of CAT-I and CAT-II is plotted against the difference between the two methods (Difference: CAT-I – CAT-II). The dotted lines indicate the 95% limits of agreements and the continuous line indicates the bias

To further compare the CAT-I and CAT-II methods, Bland-Altman analysis was applied to calculate the differences between the two assays in the presence of hemoglobin, bilirubin or lipids. This revealed that all measurements were within the 95% limits of agreement, however, some bias emerged between the two methods. While the presence of hemoglobin and lipid resulted in a negative mean bias (− 547.0 nM.min vs − 269.0 nM.min, respectively), the bilirubin presence led to a positive mean bias (370.0 nM.min) between CAT-I and CAT-II. These results were in agreement with the poor correlation in the presence of these endogenous substances (r = 0.6166 vs 0.42 vs − 0.6615, respectively). Furthermore, both hemoglobin and lipids appeared to have a similar small standard variation of bias (155.9 and 159.9 nM.min, respectively). In contrast, the presence of bilirubin resulted in a larger standard variation of bias (326.1 nM.min; Fig. 5). Comparison of the 3 conditions reveals that the presence of lipid results in the least difference between CAT-I and CAT-II, while the effect of bilirubin is the most variable.

Taken together, these data suggest that the CAT-I method is more disturbed by common analytical interferences such as hemoglobin, bilirubin or lipid in comparison with the CAT-II method. These results may indicate that the modified measurements in CAT-II allows a better compensation for these common interferences.

### Comparison of CAT-II measured in the Fluoroskan and on the ST®-Genesia

For the CAT-II on the Fluoroskan and ST®-Genesia measurements, the same calibration and trigger were applied, but the measurements were performed in a different manner. While the CAT-II method applied on the fluorometer is a batch-wise measurement (96 well plates) using a microplate reader (Fluoroskan Acsent), the ST®-Genesia is designed for unitary testing by using cuvettes. Thrombin generation parameters were calculated using a similar algorithm.

To assess the reproducibility and precision of ST®-Genesia, intra-assay coefficients of variation (CV) was calculated using 7 independent TG curves derived from non-treated plasma samples. For all parameters, CV% was less than 5% percent indicating a good reproducibility of the measurements (Table 2). ST®-Genesia represented a significantly lower thrombin generation potential indicated by reduced ETP and peak height (*p* = 0.0006 vs *p* = 0.0002, respectively) in comparison with CAT-II method. Interestingly a significantly lower lag time (*p* < 0.0001), but an increased time to peak (*p* = 0.0015) occurred with ST®-Genesia when compared with CAT-II method (Table 2). Despite the significant differences in lag time, it is noteworthy that the differences in lag time between the two methods is only 8 s and hence the actual relevance of this difference is debatable.

**Table 2:** Mean TG parameters and %CV measured using CAT-II and ST®-Genesia

TG parameter	CAT-II		ST®-Genesia		P-value
	Mean [SD]	CV%	Mean [SD]	CV%	
Lag time (min)	2.35 [0.04]	1.9	2.43 [0.08]	3.1	< 0.0001
ETP (nM.min)	1402 [68.32]	4.9	922.8 [14.49]	1.6	0.0006
Peak height (nM)	265.9 [9.59]	3.6	179.9 [8.77]	4.9	0.0002
Time to peak (min)	4.98 [0.04]	0.8	4.58 [0.17]	3.7	0.0015

*TG* thrombin generation; *CAT* calibrated automated thrombogram; *CV* coefficient of variation, *ETP* endogenous thrombin potential

**Fig. 6:**
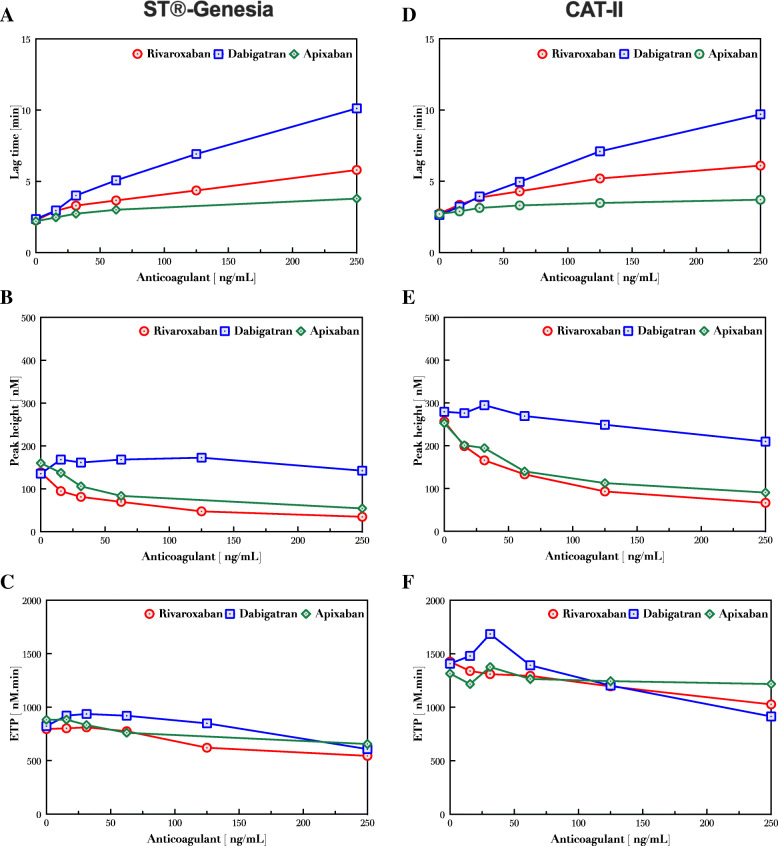
Dose-dependent effect of DOACs on thrombin generation measured by ST®-Genesia and CAT-II. Plasma samples were spiked with dabigatran, apixaban or rivaroxaban (0–250 ng/ml) and thrombin generation was measured with ST®-Genesia and CAT-II. Lag time (**A**,**D**), peak height (**B**,**E**) and ETP (**C**,**F**) were determined in thrombin generation curves obtained with ST®-Genesia and CAT-II methods

Next, we investigated the effect of DOACS on thrombin generation measured by ST®-Genesia and Fluoroskan. Plasma samples were spiked with different concentration (0–250 ng/mL) of rivaroxaban, dabigatran or apixaban followed by thrombin generation measurements. A similar dose-dependent effect appeared using both instruments indicated by increased lag time (Fig. 6A, D), decreased peak height (Fig. 6B, E) and decreased ETP (Fig. 6C, F). This similarity suggests that using the same calibration method results in highly similar outcome. Furthermore, ST®-Genesia resulted in a similar lag time, but a generally lower peak height and ETP compared with the Fluoroskan, like it was observed on the vehicle measurements. This general differences between the two methods might originate from the difference in temperature maintenance as ST®-Genesia has a better temperature control due to its complete isolation and closeness.

## Discussion

In the present study we aimed to characterize a new thrombin generation method (CAT-II) and to compare this to the widely used Calibrated Automated Thrombogram (CAT-I). The main purpose of CAT-II was to overcome the pitfalls and drawbacks of the CAT-I method. Secondly, we have compared CAT-II on the Fluoroskan and on the ST®-Genesia.

The main difference between CAT-I and CAT-II methods is the calibration that highly influences the outcome. While in CAT-I, thrombin activity required for the calibration is measured in the plasma samples using a thrombin that is in complex with α2M [1], the CAT-II method applies a buffer system to measure the thrombin needed for the calibration process. Regardless of the method of calibration, the intra-assay variation was below 5% in case of all measured parameters (lag time, ETP, peak height, time to peak) when control plasma samples were measured. This suggests a good reproducibility and that the new calibration method provides comparable results to the established CAT-I method.

However, the modified calibration method becomes more important when thrombin generation needs to be measured in samples containing DOACs. While CAT-I method can be used in plasma containing direct factor Xa inhibitors such as rivaroxaban or apixaban, it cannot be used in the presence of a direct thrombin inhibitor (e.g dabigatran) since the anticoagulant would inhibit the thrombin in the calibrator as well. Our results have shown over 97% correlation with all parameters between the results of CAT-I and CAT-II in the presence of either rivaroxaban or apixaban, albeit the correlation varied between 31 and 99% depending on the parameter in the presence of dabigatran. Only a weak correlation occurred in peak height when dabigatran was present. Peak height showed a steep dose-dependent increase in the CAT-I method while this effect disappeared in the CAT-II method. This discrepancy was in accordance with previous publications [10, 11] and the explanation can be found in the calibration method. The algorithm used in CAT-I calibration uses the initial slope of the calibration curve which is severely inhibited by the presence of direct thrombin inhibitors. In order to overcome this problem, modification of the thrombin generation assay was proposed, wherein a known concentration of AMC is measured in plasma without calibrator [12]. The measured fluorescence intensity of the known concentration of AMC is calculated back to 1 μM AMC for each sample and implemented in the algorithm thereby allowing thrombin generation measurement in the presence of direct thrombin inhibitor [12]. This method does neither rely on the effect of dabigatran in the plasma samples, nor take into account a possible difference between chromogenic substrates. In contrast, the CAT-II method described in this paper circumvents this problem as it performs the calibration in a buffer system with the combination of AMC measurement within the plasma.

The presence of direct thrombin inhibitors is not the only interfering condition, also other endogenous substances can affect the thrombin generation outcome. A high amount of triglycerides, hemoglobin or bilirubin changes the absorbance of the plasma that may interfere with laboratory outcome [13]. In order to see whether the CAT-I or CAT-II method is more reliable for such interference, plasma samples were supplemented in vitro with hemoglobin, bilirubin or lipid. This revealed no impact on the thrombin generation parameters measured with CAT-II, whilst CAT-I experienced some interference by bilirubin and lipid, but not hemoglobin content. The direct comparison of the two methods revealed the biggest variation in the presence of bilirubin. Given that patients with hyperlipidemia, or hemolysis have been described with a higher thrombotic risk [3, 14], the CAT-II method may provide a more reliable approach for monitoring these patients.

After careful characterization of the CAT-II method using Fluoroskan, we also compared it to ST®-Genesia, an automated thrombin generation method using the same calibration approach. Thrombin generation in non-treated plasma measured by ST®-Genesia resulted in low intra assay variation (< 5%) that was in agreement with previously reported data as well as the ETP and peak height values [7]. Interestingly, ST®-Genesia resulted in generally lower thrombin generation parameters in plasma from healthy volunteers in comparison to the CAT-II method applied on the Fluoroskan. This difference might originate from the difference in temperature control as ST®-Genesia is a fully closed system which allows more precise temperature control (37 °C) while the plate reader may have a less stable temperature control. It has been shown that increasing temperature results in lowering of the thrombin generation curve displayed by lower ETP and increased lag time [15–17].

Similarly, a study conducted in liver transplant patients showed an increase in TG parameters when it was assessed by CAT-I and compared to ST®-Genesia [18]; however, this is in discrepancy with a previous publication showing an unchanged ETP and increased peak heights in healthy controls measured by ST®-Genesia in comparison to CAT-I [19]. Furthermore, Talon et al. has used the ST®-Genesia reagents in the CAT-I method and found a decreased ETP and peak height compared to the ST®-Genesia [19]. This is in agreement with our data showing that the direct comparison of CAT-I with ST®-Genesia resulting in a generally lower TG using the latter method.

Next, we investigated the effect of DOACs on thrombin generation parameters that appeared to be highly similar between ST®-Genesia and CAT-II on Fluoroskan. However, overall lower values were measured in the ST®-Genesia measurements with a difference comparable to the one seen in non-treated samples mentioned above. A similar dose-dependent effect has been reported in patients with DOACs when a different trigger, STG-Drugscreen was used to trigger thrombin generation [20]. Considering that in current clinical practice, monitoring of patients treated with a DOAC is not sufficient to estimate the degree of the anticoagulant effect [21], thrombin generation could provide a good alternative. However, the association between suppressed thrombin generation and clinical outcome has to be carefully investigated.

## Conclusions

Our results show that the new CAT-II and ST®-Genesia method provides a valuable tool to measure thrombin generation in samples with either direct anticoagulants or elevated levels of endogenous substrates like hemoglobin, lipid or bilirubin. Given that our experiments have been performed in a low number of healthy donors, future studies need to take place in order to evaluate the clinical relevance of this method.

## Data Availability

Original data are available on request.

## Abbreviations

TG: Thrombin generation
CAT: Calibrated automated thrombogram
DTI: Direct thrombin inhibitor
DOACs: Direct oral anticoagulants
AMC: 7-amino-4-methylcoumarin
α2M-T: Alpha-2-macroglobulin-thrombin complex
SD: Standard deviation
CV: Coefficient of variation
ETP: Endogenous thrombin potential
TF: Tissue factor
PL: Phospholipid
